# Disruptive Child Behavior and Income Inequality: Examining Long-term Maintenance of Family Income Levels in Families Receiving Parent-Training

**DOI:** 10.1007/s11121-025-01830-x

**Published:** 2025-08-13

**Authors:** Lea Tangelev Greve, Hanne Nørr Fentz, Tea Trillingsgaard

**Affiliations:** Developmental Psychology, Department of Psychology and Behavioral Sciences, Aarhus University, Bartholins Allé 11, 8000 Aarhus C, Denmark

**Keywords:** Disruptive child behavior, Contextual inequality, Parent training, Long-term effects, Family income, Quasi-experimental study

## Abstract

This study explores long-term maintenance of family income levels in families receiving parent training for disruptive child behaviors. We use data from the Danish implementation of the Incredible Years Parent training (IYPT) across 21 municipalities from 2012 to 2019. Utilizing a quasi-experimental design with matching of a subsample of 707 out of the 1229 families from the Danish IYPT sample with 690 control families drawn from the national registers, we compare annual disposable family income in intervention families with the background population and control families from 2 years before to 4 years after pretest. Our findings reveal that intervention families and control families had significantly lower annual disposable family income than the background population families across all time points. For intervention families, the financial gap from the background population families widened from USD 11,268 to USD 16,694 from the first to the last time point. Adjusted regressions comparing intervention families to control families found a small but significant financial gap, so that intervention families had USD 2189 less to their disposal per year from the first time point and USD 7596 less per year at the last time point. These results suggest that intervention families faced increasing financial strain from years before up to 4 years following the IYPT, both in relation to the general Danish population and to the matched control of socioeconomically similar families across an 8-year span. We suggest that this could reflect continued stress and disruption of work schedule due to child behavior problems. Regardless of the underlying mechanism, these findings underscore the importance of considering the long-term economic contexts of families with disruptive child behaviors. Societal strategies that address both parenting challenges and broader contextual inequalities may be needed to support healthy child development.

Disruptive child behavior includes threatening, deceitful, hurtful, spiteful, or anti-social actions (Bierman & Sasser, 2014). Behavioral disorders are characterized by abnormally high frequencies or severity of such behaviors considering the child’s age (Tandon & Giedinghagen, 2017). Attention deficit hyperactivity disorder (ADHD), oppositional defiant disorder (ODD), and conduct disorder (CD) are three mental disorders all characterized by high levels of disruptive behaviors (American Psychiatric Association, 2013). Up to 60% of children with ADHD also qualify for a behavioral disorder (American Psychiatric Association, 2013), and ODD and CD collectively represent more than a third of all mental disorders diagnosed in children worldwide (Polanczyk et al., 2015). A meta-analysis estimated European prevalence rates for ODD (1.9%), CD (1.5%), and ADHD (2.9%) (Sacco et al., 2024), highlighting their global public health significance.

Disruptive child behaviors are known to place considerable strain not only on the child but also on his or her caregivers (Christenson et al., 2016). Child behavioral disorders impose financial and labor-market burden comparable to chronic physical child disorders like epilepsy and diabetes, including costs of specialized care and reduced parental work hours (Busch & Barry, 2007). Behavioral disorders disrupt parents’ work life more than other child health conditions (Busch & Barry, 2007). In an online survey with 2326 caregivers of children receiving ADHD medication, caregivers were asked about the impact of their child’s ADHD on their work life (Fridman et al., 2017). Fridman et al. (2017) found that on average, caregivers reported missing 3.9 h of work during the past month due to their child’s ADHD, 28% of caregivers reported having had to change jobs or modify their work hours because of their child’s ADHD, and 6% of caregivers reported having to quit their job because of their child’s ADHD. Brennan and Brannan (2005) found that the severity of externalizing child symptoms was directly related to the experience of ‘caregiver strain from missing work or neglecting duties’ in a survey sample of 2858 parents of children aged 5–17 years in the USA. These findings support the hypothesis that disruptive behaviors challenge parents’ work productivity and could ultimately be linked to lowered family income levels over time.

## Spill-over Effects on Income from Behavioral Parent-Training

Behavioral parent-training programs have consistently been found to reduce disruptive child behaviors and dysfunctional parenting behaviors and increase positive constructive parenting behaviors (Buchanan-Pascall et al., 2018). They have also been found to have beneficial maintained small-moderate effects on parenting stress and parenting cognitions (e.g., parenting self-efficacy, parenting satisfaction, or parenting self-competence) (Barlow et al., 2012; Colalillo & Johnston, 2016; Weber et al., 2019). The Incredible Years Parent training program (IYPT) is an evidence-based and widely implemented intervention aimed at preventing and treating disruptive child behaviors in children and youth (Gardner & Leijten, 2017; Menting et al., 2013; Webster-Stratton, 2001). The IYPT targets dysfunctional parenting behaviors building on theoretical understanding that negative cycles of disruptive child behaviors and coercive parenting behaviors tend to worsen over time and cause dysfunction in the family and parent–child relations (Dishion, 2014; Patterson, 2015). Randomized controlled trials have found the IYPT to effectively reduce disruptive child behaviors in both university (Webster-Stratton, 1984; Webster-Stratton & Hammond, 1997; Webster-Stratton et al., 2004) and community settings (Axberg & Broberg, 2012; Karjalainen et al., 2019; Larsson et al., 2009) with small-moderate effect sizes (Gardner & Leijten, 2017; Menting et al., 2013). Benchmark studies have found that the IYPT reduces parent-reported disruptive child behaviors in Danish children at a comparable or higher level of effectiveness compared to gold-standard RCTs (Greve et al., 2023; Trillingsgaard et al., 2014) with large effect sizes. It was also found that the Danish implementation of the IYPT was equally effective at reducing child behavior problems across levels of accumulated socioeconomic risk and socioeconomic family background (Greve et al., 2024).

Given the impact of disruptive child behavior on parental work life and the effectiveness of parent training, spill-over effects on family income may be expected. However, findings in this area are missing. A study of 42 children found that the Triple P program improved parental work-related self-efficacy, with effects maintained at follow-up (Martin & Sanders, 2003). In this study, there were also significant reductions of work stress and improved adjustment for parents in the treatment group from pretest to follow-up, but not from pre-posttest, indicating that some program effects may develop over time rather than immediately after the intervention (Martin & Sanders, 2003). Such findings support a hypothesis of positive spill-over effects from the effects of parent training to maintained work-related productivity and ultimately maintained levels of family income over long-term follow-ups. No prior studies have tested this hypothesis using long-term objective family income measures.

## Theoretical Background

Families with child behavior problems face financial strain due to missed work, reduced hours, and increased caregiving demands. Theory explains the reverse type of causality, that is, how the experience of financial strain in a family may lead to increases in disruptive child behaviors. The Family Stress Model by Conger et al. (2010) posits that parents experiencing socioeconomic stress tend to decrease their investment in their child both in terms of emotional engagement, time, and money. According to this model (Masarik & Conger, 2017), socioeconomic stress in the family is linked with higher levels of parental psychological distress and interparental conflict. Parents’ psychological functioning and level of interparental conflict are both linked to negative changes in parenting behaviors, such as being less sensitive and responsive towards the child, exercising more harsh and inconsistent parenting, and investing a lower amount of time with the child (Masarik & Conger, 2017). Conger and colleagues have tested their model and found support for the hypothesized mechanisms in several longitudinal observational studies (see review in Masarik & Conger, 2017).

To sum up, according to the Family Stress Model, parents’ perceived socioeconomic stress could lead to lower investment in their child in terms of time, money, and emotions. There is ample evidence from developmental psychology studies to show that harsh, inconsistent, insensitive, or uninvolved parenting behaviors can lead to the onset or worsening of child behavior problems (Conger et al., 2015; Dishion, 2014; Patterson, 2015). The pathways of the Family Stress Model have found support in longitudinal studies across different ethnic groups, countries, and family structures (Masarik & Conger, 2017). For example, Neppl et al. (2016) found that in a sample of 273 families, across three time points, and using both observational and self-report data, economic strain led to parental distress and couple conflict, which led to harsh parenting and child problem behaviors, even when controlling for early disruptive child behaviors.

In a recent study of a large British cohort of 2399 children and their parents, Piotrowska and colleagues (Piotrowska, et al. 2023) found that disruptive child behaviors mediated the effects of low family income on parental functioning, so that parental functioning suffered from financial strain only in families with child behavior problems. This finding differs somewhat from the suggested mechanism in the Family Stress Model in which financial strain leads to dysfunctional parenting behaviors that lead to increases in child behavior problems. The authors suggest that children in low-income families tend to experience concomitant higher levels of disruptive child behaviors that could contribute to increased parental stress and inhibit parental functioning (Piotrowska et al., 2023).

In sum, this theory and research point to consistent links between financial strain, lowered parent functioning, and child disruptive child behavior, while the pathways between these three factors are likely complex. Inspired by the developmental cascade model of aggressive child behavior, as described by researchers such as Dishion (2014), Doty et al. (2017) proposed that positive changes may also function as developmental cascades, spreading through the family system over time. They suggested that this process could serve as a mechanism for building resilience within families following parenting interventions. Their conceptual model illustrates how improvements in effective parenting behaviors and parental self-efficacy can have positive ripple effects on other life domains, such as health and peer support systems. These improvements, in turn, may foster positive emotions and reduce stress, ultimately strengthening both the family system as a whole and its individual members in the long term.

Based on the above literature review, we argue that changes in either parent functioning, child disruptive behavior, or finances will likely be associated with changes in the others. Thus, while economic stress can lead to increases in disruptive child behaviors due to negative changes in parenting behaviors, a negative developmental process suggested by the Family Stress Model, parent training may initiate a developmental cascade of positive processes where changes in disruptive child behaviors positively affect parents’ work productivity and ability to maintain family income levels. Because the Danish implementation of the IYPT parent training has been found to successfully reduce child disruptive behavior in participating families with large effect sizes (Greve et al., 2023) across socioeconomic risk levels (Greve et al., 2024), we would expect positive spill-over effects on maintained family income levels in the years following the intervention.

### Aims and Hypotheses

In this study, we tested two hypotheses. First, we expected intervention families (i.e., families who participated in the IYPT aimed at reducing disruptive child behaviors) to have lower annual disposable family income levels compared to the background population over time.

Second, we expected intervention families to show positive average spill-over effects on the maintenance of disposable family income levels in the years after receiving the intervention as compared to (matched) control families. In other words, we expected smaller gaps between family income levels in intervention families versus background population families, and relatively larger gaps between family income levels in control families versus background population families. See section “Analytic Approach” for a detailed description of the matching procedure.

## Methods

### Procedure

For the main study (see Greve et al., 2023), data were generated by a centralized data collection during the national implementation and roll-out of the IYPT in Denmark during the years 2012–2019. Thus, 21 Danish municipalities contributed with data on 188 parent-groups treating 1229 children. Families entered the IYPT through self-referral, formal referral through social services, or prompted by a professional (e.g., a health nurse, day care or school staff, family physician), depending on local implementation strategies (Servicestyrelsen, 2011). Families then all received visitational meetings including a screening of the severity of disruptive child behaviors, information on the program, and evaluation of parental motivation for participation before, if relevant, being offered the intervention. The screening consisted of the parent(s) filling out the Eyberg Child Behavior Inventory (Eyberg & Pincus, 2010; see “Measures” for further details). Some municipalities used the clinical cut-off values as a strict inclusion criterion, while other sites used the ratings to inform their clinical decision to include families or not. Thus, families with non-clinical screening results may have been invited to participate if the visitation meeting led visitators to believe that child disruptive behaviors and parental management were among the key issues in the family. Similarly, families with clinical screening results may not have been invited to participate if the visitation meeting led staff to believe that other factors—such as physical abuse or other obvious external stressors—likely explained the high level of disruptive behaviors and would hinder family participation in the program or called for some other intervention. The screening parent-report is also the pretest report for the current study. Posttest surveys on the parent-reported frequency and severity of disruptive child behaviors were collected at the final group session. Follow-up waves at 3, 6, and 12 months after posttest were planned, but retention rates past posttest were poor. This meant that only pre-to-posttest survey data can be used for statistical purposes. The low retention rates beyond posttest most likely do not reflect participant engagement in the intervention but may be explained by a lack of procedures for long-term data collecting. Thus, data collection practices at most sites ended at posttest, as this was carried out as a natural part of running the parenting groups. We refer to Greve et al. (2023) for further details on procedure.

### Participants

For the current study, inclusion criteria for intervention families were (1) being among the 1229 Danish families represented in the Danish IYPT implementation data, (2) having available data on the unique personal identifier of the child and at least one biological parent in the national registers available in this study (*n* = 31 excluded), (3) having available data on the year of receiving the intervention (*n* = 6 excluded), and, finally, (4) having available data on annual disposable family income for all time points. This approach led to a sample size of 842 intervention families across 20 municipalities. The time points were defined as follows: 3 years prior to pretest (baseline), 2 years prior to pretest (Time_−2_), 1 year prior to pretest (Time_−1_), the year of pretest (Time_0_), 1 year after pretest (Time_+1_), 2 years after pretest (Time_+2_), 3 years after pretest (Time_+3_), and 4 years after pretest (Time_+4_).

Background population families were drawn from the Danish national registers and included families with one child between the ages of 2 and 12 years during the years 2012–2019 who had available data on annual disposable family income across all time points. This led to a comparison sample of 292,966 background population families. Background population families were assigned a random year of pretest from 2008 to 2016 to serve as their Time_0_ to allow for a similar period of comparison relative to the child’s age. For example, a child was assigned the year 2010 as Time_0_. At Time_0_, this child was 4 years old. Thus, in our analyses, the family income in this particular child’s family will be analyzed from baseline (2007, where the child was 1 year old) to 2014 (where the child was 8 years old). Because the population from which background population families were drawn was so large, this procedure ensured that large numbers of children of each of the ages 2 through 12 were followed on their disposable family income over 8 consecutive years, similar to the intervention families.

Control families were drawn from a subgroup of 97,605 families from the background population living in a municipality which never offered behavioral parent-training. Of these, statistical matching on family characteristics created a matched control group of 690 control families (see section “Analytic Approach” for details on matching procedure). Below, Table 1 shows the baseline descriptive statistics for intervention families, background population families, and for control families.

**Table 1:** Baseline characteristics of the background population, the intervention families, and their matched controls

	Background population	Intervention families	Control families
Variable	*n* = 292,966^a^	*N* = 842	*n* = 690
Child age *M* (SD)	4.0 (1.4)	3.5 (1.9)	3.6 (1.4)
Boys *n* (%)	150,204 (51.3)	573 (67.6)	442 (64.1)
Child psychiatric history *n* (%)	321 (0.1)	11 (1.3)	< 5 (< 1)
Social services *n* (%)	917 (0.3)	27 (3.2)	< 5 (< 1)
Single parent household *n* (%)	45,420 (15.5)	247 (29.1)	200 (29.0)
Low income^b^ *n* (%)	40,209 (13.7)	192 (22.6)	162 (23.5)
Maternal age *M* (SD)	34.5 (5.0)	32.1 (5.8)	30.8 (6.1)
Poor maternal health^c^ *n* (%)	28,149 (9.6)	144 (17.0)	13 (1.9)
Maternal psychiatric history^d^ *n* (%)	1051 (0.4)	37 (4.4)	9 (1.3)
Mother divorced *n* (%)	18,977 (6.5)	80 (9.4)	49 (7.1)
Low maternal education^e^ *n* (%)	72,079 (24.6)	327 (38.6)	293 (42.5)
Teen mother *n* (%)	2979 (1.0)	37 (4.4)	26 (3.8)
Maternal unemployment *n* (%)	30,899 (10.6)	186 (21.9)	172 (24.9)
Mother immigrant^f^ *n* (%)	39,433 (13.5)	70 (8.3)	96 (13.9)
Annual family income in DKK *M* (SD)	420,643.1 (156,380.9)	366,454.2 (155,026.5)	369,948.5 (169,679.4)

Baseline is 3 years before participation in the treatment

^a^The background population consists of all families living in Denmark and having a child aged 2–12 years between the years 2012 and 2019

^b^Low income refers to the child living in a household with an annual disposable income below the 10th percentile family income in the background population

^c^Poor health refers to having more annual contacts with public somatic health care services (i.e., in- and outpatient clinic visits, emergency room visits, on-call doctor’s contacts, and general physician contacts) than 90% of mothers in the background population

^d^Psychiatric history refers to having one or more formal mental health diagnoses related to disruptive behaviors (e.g., oppositional defiant disorder, conduct disorder, or attention deficit hyperactivity disorder for children and substance abuse disorder, antisocial personality disorder, or attention deficit hyperactivity disorder for mothers) and/or taking prescription medication for attention deficits within the past year

^e^Low education refers to having no education beyond the compulsory 10–11 years of Danish primary and secondary school

^f^Immigrant refers to being formally registered as an immigrant or a direct descendant from immigrants

### Intervention

Families received either the preschool or the school-age version of the well-established, group-based parent-training program Incredible Years basic (targeting disruptive behaviors in children aged 2–7 or 8–13 years, respectively) (Webster-Stratton, 2001). The program is manualized and was developed from the 1980 s and onwards to train parents in the use of efficient and non-coercive ways of discipline towards aggressive or destructive child behaviors and to model and reward prosocial and positive behaviors during parent–child interactions (Webster-Stratton, 2001). Sessions include a mix of information on child development, discussion of a video vignette showing a parent–child interaction, sharing of experiences among the parents, role play, a review of last week’s homework (parents implement new skills between sessions and discuss successes and challenges), and a walk-through of homework for the next session (Webster-Stratton & Herbert, 1993). Every parent group included the parent(s) of five to six children and met for 12–14 weekly 2-h sessions led by a trained group leader with a relevant clinical background (i.e., a health nurse, family therapist, or psychologist). Group leaders received in-person training in a 3-day workshop where experienced, certified trainers role-modeled the approach of the program applying key methods, while going through the program manual, theory and strategies such as modeling of prosocial behaviors, rewarding prosocial behaviors, and ignoring unwanted behaviors (Webster-Stratton & Reid, 2011). To achieve formal group leader certification, group leaders additionally needed to complete two IYPT groups, including video supervision, the use of manuals and fidelity checklists for each session, and concurrent participant evaluations (Servicestyrelsen [Danish Social Services Agency], (Servicestyrelsen. 2011)). For many group leaders in this study, the data were collected for the two first parent groups run by a group leader as part of their certification process, meaning that these group leaders were actively tracking and receiving concurrent feedback on their fidelity in terms of sticking to the manual, applying the key formats and contents of the program, and facilitating a constructive group dynamic (Servicestyrelsen, 2011). They also video filmed an entire session from two different parent groups and sent it to the program developers who then delivered individual, online supervision, and they were offered regular peer coach supervision during the year from more certified group leaders from other municipalities (Servicestyrelsen, 2011). Unfortunately, we do not have access to fidelity data. The focus of the current study is solely on the preschool and school-age versions of the parent-training program BASIC.

## Measures

### Background Characteristics of the Family

Through the unique civil registration number ascribed to every individual born in Denmark since 1969 in the national registers, it is possible for researchers to link data on individuals to their family members, to access data on a range of different domains (e.g., health, demography, and economy), and to track changes both back and forth in time (Thygesen et al., 2011). Importantly, at the point of researcher access, any register-based data are strictly pseudonymized and cannot be traced back to any actual civil registration number (Statistics Denmark, 2014). Descriptive register–based data for this study included information on year of birth (child and mother), gender (child), formal psychiatric diagnoses (child and mother), retrieval of prescription drugs for attention deficit disorders (child and mother), social service referral to preventive family intervention (child), household type (i.e., single-parent or two-parent household; child), somatic health care contacts (e.g., yearly contacts to general physician, emergency rooms, hospitalizations; mother), marital status (i.e., married, divorced, or separated; mother), employment status (i.e., being employed or not; mother), highest educational status (i.e., having education beyond primary or secondary (= compulsory) school; mother), and immigration status (i.e., mother was born in Denmark or not; child).

### Annual Disposable Family Income

This measure is in Danish crowns, DKK (USD 100 = DKK 695.39) and an aggregate value computed for administrative purposes in the Danish tax system. The variable is calculated on a family level and thus is the same for every household member within the same year. As such, annual disposable family income is not directly representative of any individual’s actual (yearly) income, but a measure meant to gauge, relative to other families, how much money the family has at their disposal for saving and spending every year after paying for a range of fixed expenses such as housing and taxes (Statistics Denmark, 2022). Data related to annual disposable family income is collected annually and validated by both the Danish authorities and the individual taxpayer. These data are thus considered to be complete (i.e., to cover the entire population), to be of “very good” quality, and to hold no sampling errors or risk of bias (see Statistics Denmark, 2022, p. 9).

## Analytic Approach

Preparation and merging of survey data and register-based data was performed using the statistical software SAS/STAT 13.1, while remaining data management, preprocess matching of intervention families to control families, and analyses were performed using STATA version 15.0. We managed extreme outliers by trimming the families with the top and bottom 1% most extreme values of family income. Trimming was performed pre-matching on the combined sample of the Danish population and the IYPT families.

### Matching Procedure

The test of causal inference in intervention studies requires an experimental design. In real-life community settings, randomization of patients or clients into an intervention or a control condition may not be feasible once an intervention is implemented and available for all citizens; for example, in a defined geographical area, such as a municipality. In the current study, the Danish implementation, roll-out, and data collection of the IYPT in 21 municipalities unfolded with no researcher involvement and no control group. Matching on the propensity scores aims to reduce bias by obtaining a matched sample of individuals, not necessarily similar to the treated sample on all covariates, but on propensity for selection into intervention, described as ‘balancing on covariates’ (Rosenbaum & Rubin, 1983). We used a logistic regression model to calculate propensity scores. We matched intervention families to control families from a subgroup of the families in the background population living in municipalities which never offered behavioral parent-training programs, on the following covariates measured at baseline (3 years prior to pretest): child age, gender, household type, and parental age, educational level, employment status, contact with the somatic healthcare system, psychiatric history, as well as baseline annual disposable family income. Matching resulted in 707 out of 842 possible matches for the intervention families to 690 control families out of 97,605 background population families from non-parent-training municipalities (further details on the matching procedure, covariate balance diagnostics, and model specifications can be found in the Appendix, and STATA code is available from the authors upon request). The matching procedure led to acceptable standardized mean differences (all <|.10|) and standardized differences in variance (at least approaching 1.00 and below 2.00) between matched and unmatched observations (see Appendix). Baseline differences between matched controls, matched intervention participants, and population controls can be seen in Table 1 above. The STATA package KMATCH (Jann, 2017) was used to calculate propensities and to perform the matching.

### Main Analyses

Our first hypothesis was that there would be a baseline income gap between intervention families (*n* = 842) and the background population families (*n* = 292,966). We examined this by conducting independent samples *t-*tests comparing the mean annual disposable family income of the intervention families to that of the background population families across 8 years: from baseline to Time_+4_.

Our second hypothesis was that receiving the IYPT would lead to a positive average long-term spill-over effect on the annual disposable family income of intervention families compared to control families, so that intervention families would have higher annual disposable income than control families in the years following intervention. After matching, we tested our second hypothesis by conducting adjusted regression analyses to calculate the average spill-over effect on the intervention families, i.e., the mean difference in annual disposable family income between the intervention families with a match (*n* = 707) and the control families (*n* = 690) on all time points after baseline (i.e., Time_−2_ to Time_+4_). Adjusted regression works by fitting a line of best fit to the intervention families and control families separately and then using the slope of the control families to estimate potential outcomes for the intervention families (Ho et al., 2007). The average spill-over effect on the intervention families, then, is the difference between the observed and the estimated mean outcome for all intervention families, i.e., the difference between their actual income after receiving the IYPT and their hypothetical income as predicted by the line of best fit of the control families. We used the KMATCH module in STATA (Jann, 2017) to conduct the regression adjustment with the same covariates used for matching included as control variables (measured at baseline) in the regression models and estimated bootstrapped standard errors across 100 draws to obtain 95% confidence intervals and significance levels.

### Sensitivity Analyses

One (conservative) approach to sensitivity tests following a matching procedure is to test for sensitivity to unobserved confounders using Rosenbaum’s bounds (DiPrete & Gangl, 2004). It entails using Wilcoxon signed rank tests to calculate *p*-values for our results (the spill-over effect of the intervention on annual disposable family income at each of the seven time points after baseline) at different levels of potential unobserved bias. The test shows at what level of unobserved bias (i.e., at what level of *Γ*) our results would cease to reach significance *in a worst-case scenario*, where the odds ratio of being assigned to the intervention or the control condition differ by *Γ*, *and* where the association between the unobserved variable and the outcome explains the majority of the variance in outcome between every pair of matched treated and control cases (DiPrete & Gangl, 2004). Thus, the Rosenbaum bounds inform on the robustness of the result against unobserved confounders depending on how strongly such a confounder is associated with both selection and outcome, and results in a conservative “worst-case” estimate of “how strongly must an unobserved confounder be (i.e., at what value of *Γ*) to change our results from statistically significant to non-significant.” We used the Rbounds (Gangl, 2004) STATA module for this.

## Results

**Fig. 1:**
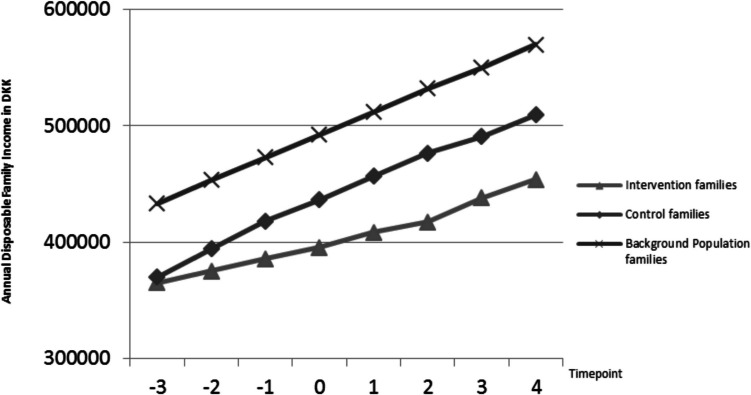
Mean annual disposable family income in intervention, control, and background population families across 8 years

Below, Fig. 1 plots the annual mean disposable family income of intervention families, families in the background population, and control families across the eight time points, including baseline.

Across seven timepoints after baseline, from 2 years prior to the intervention to 4 years after, independent samples *t-*tests comparing the annual disposable family income of the intervention families to that of families in the background population found a financial gap, so that intervention families had less money at their disposal every year. The difference between groups ranged from DKK 78,418 (USD 11,277) at Time_−2_ to DKK 116,089 (USD 16,694) at Time_+4_; thus, the income gap steadily widened over the period. A similar pattern was found when comparing intervention families to control families, and again with intervention families having less money at their disposal every year, although with a slightly smaller widening of the gap over time, ranging from a difference of DKK 78,901 (USD 11,384) at Time_−2_ to DKK 93,266 (USD 13,412) at Time_+4_ (see Table 2).

**Table 2:** Results: annual disposable family income across groups from 2 years prior to intervention to 4 years after

	Time						
Group	− 2 years	− 1 year	Year 0	+ 1 year	+ 2 years	+ 3 years	+ 4 years
BP income *M* (*SD*)	453,583 (184,858)	473,010 (195,558)	492,332 (206,286)	512,010 (218,823)	532,164 (230,843)	549,757 (243,143)	569,940 (256,962)
C income *M* (*SD*)	374,682 (161,805)	391,231 (164,726)	405,118 (174,743)	425,139 (178,795)	442,527 (192,564)	459,148 (204,910)	476,674 (215,747)
I income *M* (*SD*)	375,165 (180,522)	385,952 (178,270)	395,562 (189,415)	408,486 (193,150)	417,411 (198,457)	438,248 (209,954)	453,851 (219,916)
I vs. BP^a^	*t* = 11.3, *p* =.000	*t* = 11.8, *p* = .000	*t* = 12.5, *p* = .000	*t* = 12.6, *p* = .000	*t* = 13.2, *p* = .000	*t* = 12.2, *p* = .000	*t* = 12.0, *p* = .000
C vs.BP^a^	*t* = 11.2, *p* = .000	*t* = 11.0, *p* = .000	*t* = 11.1, *p* = .000	*t* = 10.4, *p* = .000	*t* = 10.2, *p* = .000	*t* = 9.8, *p* = .000	*t* = 9.5, *p* = .000
I vs.C^b^	− 15,043 *p* = .007 CI 95% [− 26,044; − 4044]	− 20,831 *p* = .001 CI 95% [− 32,747; − 8,917]	− 25,980 *p* = .000 CI 95% [− 40,462; − 11,499]	− 33,717 *p* = .000 CI 95% [− 49,388; − 18,044]	− 42,023 *p* = .000 CI 95% [−59,940; − 24,106]	− 39,618 *p* = .000 CI 95% [− 57,920; − 21,317]	− 42,077 *p* = .000 CI 95% [− 62,482; 21,672]

*BP* background population *n* = 292,966, *C* control families *n* = 690,* I* intervention families *n* = 707

Income refers to annual disposable family income in DKK (100 USD = 695.5 DKK)

^a^Results from independent samples *t*-tests, df = 300,187

^b^Results from adjusted regression analyses

After matching intervention families to control families, we addressed our second hypothesis by conducting adjusted regression models to compare annual disposable family income in intervention families to that of control families measured at all seven timepoints after baseline. In every model, we controlled for the baseline level of healthcare use, child gender, child age, maternal age, annual disposable family income, long-term sickness absence, single parenthood, unemployment, low-educational level, and maternal psychiatric history related to disruptive behaviors. These results are also reported in Table 2 above along with their bootstrapped 95% confidence intervals and associated significance levels. Although intervention families and control families had been matched on baseline income, health, and socioeconomy, there was an income gap between the two groups across all time points in the analysis. This gap meant that intervention families consistently and increasingly had less money at their disposal every year from before receiving intervention to after compared to the control families. This difference ranged from DKK 15,233 (USD 2191) at Time_−2_ to DKK 52,853 (USD 7600) at Time_+4_.

### Sensitivity Analyses

Rosenbaum’s bounds are considered to be a conservative measure of ‘worst-case scenario’ sensitivity to confounding of adjusted regression (DiPrete & Gangl, 2004). We calculated Rosenbaum’s bounds for every adjusted regression model to determine the critical strength (*Γ*) at which potential unobserved confounders would affect our results to a degree that would make them statistically non-significant. Consistent with the pattern that the financial gap between intervention families and control families was smaller at earlier timepoints and widened over time, we found that the robustness of our results towards unobserved confounders of the relationship between intervention condition and annual disposable family income increased over time. In practical terms, this means that if unmeasured confounders were to increase the odds of receiving intervention by just 4% more than the observed distribution, the result at Time_−2_ would no longer be statistically significant. This suggests a relatively low level of robustness against unmeasured confounding. At Time_+2_, the timepoint with the most robust results, the result would remain significant even if unmeasured confounders could increase the odds of receiving intervention by up to 28% more than the observed distribution.

### Post Hoc Analyses

To further our understanding of our results, we opted to conduct post hoc subgroup analyses to see whether intervention response affected change in disposable family income over time for intervention families. We did this aiming to find further support for either the interpretation of our findings that parent-training did not affect disposable family income or the interpretation that parent-training affected disposable family income in families who responded well to the intervention. We conducted our post hoc subgroup analyses on annual disposable family income across all seven timepoints after baseline. We created a binary predictor variable indicating intervention response. Intervention response was defined as both experiencing reliable pre-to-posttest change according to Jacobson et al.’s (1984) equation and experiencing a pre-to-posttest reduction of 0.5*SD in disruptive child behaviors as measured on the Eyberg Child Behavior Inventory (Eyberg & Pincus, 2010). Non-response was defined as both not experiencing reliable change and experiencing a reduction of less than 0.5*SD in disruptive child behaviors. This resulted in *n* = 333 responders and *n* = 133 non-responders among the intervention families. Responders compared to non-responders did not differ on baseline annual disposable family income, *t*(464) = − 1.6, *p* = 0.109. Linear multiple regressions controlling for baseline maternal health, age, income, long-term sickness absence, household type, employment status, educational level, and psychiatric history (i.e., the same control variables used in our main analyses) found that intervention response predicted annual disposable income only at Time_−2_, *F*(10,455) = 97.7, *p* = 0.000, so that responders had DKK 23,650 (USD 3400) more at their disposal that year than non-responders, the regression coefficient being significant at *p* = 0.01. Intervention response did not predict differences in annual disposable family income at any other timepoints, *p*-values ranging from 0.077 to 0.231.

## Discussion

This study is the first to examine long-term maintenance of family income levels in the years following participation in parent-training. In support of our first hypothesis, we found that intervention families had significantly lower annual disposable income compared to the general population across all timepoints from 3 years prior to pretest (baseline) to 4 years after pretest. Furthermore, the financial gap between intervention families and the background population widened by 48% from Time_−2_ to Time_+4_. A similar pattern was observed for control families, although the gap widened to a lesser extent by 18% over the same period. Second, we expected positive spill-over effects from parent-training, such that intervention families would show smaller declines in annual family income levels compared to control families in the years after receiving the IYPT. Contrary to this expectation, intervention families experienced greater declines in annual family income levels over time compared to control families. As a result, the income gap between intervention families and control families was 3.5 times larger at Time_+4_ than it was at Time_−2_. Our findings suggest that families participating in IYPT faced greater financial strain than both background and matched control families, with no evidence of positive spill-over effects on income. The following discussion considers two competing interpretations of the widening gap in family income between the intervention and the control families.

If we suppose that participating in the IYPT, contrary to our expectation, had a negative influence on family income changes, the Family Stress Model (Masarik & Conger, 2017) may provide a theoretical explanation of how some of the behavioral changes in parenting promoted by the intervention may have unintended negative secondary long-term consequences for family income besides the intended positive primary effects on disruptive child behaviors. Part of the IYPT learning goals concerns increasing parental engagement with their child in terms of spending more time in shared activities and engaging in the child’s emotional life, for example, through child-directed play, and sharing positive emotions of appreciation and affection with their child to improve the parent–child relationship quality (Webster-Stratton & Reid, 2011). The Family Stress Model would frame such behavioral changes as *increased parental investments*. Hypothetically, some parents may decide to implement larger changes in their daily life and priorities along these lines, for example, changing into part-time positions, taking leave, abandoning work-intense and highly paying careers, or generally spending more time or energy with their child.

However, it seems unlikely that such changes in parental investment explain the widening income gap between intervention families and control families, for two reasons. First, our post hoc analyses generally did not support a link between intervention response and predicted disposable family income following the intervention. Second, the gradual widening of the financial gap began prior to and continued without changing its course in the years following the intervention.

We propose that pre-existing differences, rather than IYPT participation, explain the widening income gap. Matching, as a quasi-experimental method, is a valuable alternative when a causal design is not possible, but it leaves a risk of bias from unobserved variables. The most important variable that is unobserved in the matched control group is the occurrence of disruptive child behaviors in the family, as this information was not available from the registers. While our matching procedure lived up to conventional matching diagnostics (e.g., Markoulidakis et al., 2022), we must leave open the possibility that this or other variables outside of the matching procedure were driving the widening gap in family income over time, limiting the strength of causal conclusions. Some baseline differences between the intervention and control group also did remain after the matching procedure, but since we controlled for them in our analyses, this does not account for the gap we find between family income in intervention families and control families.

Thus, an alternative interpretation of our results is that the occurrence of child disruptive behavior in a family continues to incur financial strain on families regardless of the receipt of parent training (Busch & Barry, 2007). Over time, this leads to a smaller increase in annual disposable family income as compared to a population of families with similar background characteristics, but who do not have to cope with the emotional strain of dealing with, caring for, and supporting a child with behavior problems. Sensitivity tests did imply that results would be sensitive to the impact of unobserved confounders related to both selection and outcome in the case where such a confounder increased the odds of receiving the intervention with more than 28%. In the event of disruptive child behaviors in a particular family acting as a confounder, this is quite likely the case.

It should be noted that there is limited knowledge of group leader fidelity and parent attendance, and that there was some variation in screening procedures across the participating sites. While this limits the extent of causality claims on program effectiveness, a previous benchmark study considering these limitations has found that the Danish implementation of the IYPT was successful in reducing parent-reported disruptive child behaviors compared to European gold-standard RCTs (Greve et al., 2023). As there was no difference in results according to treatment response, it seems unlikely that the lack of spill-over effects of parent training on long-term family income is due to attrition or fidelity issues. Previous IYPT research has demonstrated the program to have moderate effects on disruptive child behavior (Menting et al., 2013), but effects have generally not been found to generalize from the home to the school (Drugli & Larsson, 2006; Karjalainen et al., 2021). This may suggest that while parents observe notable changes in their child’s behaviors at home, significant behavioral problems may persist in other contexts. This may lead to maintained family stress levels and parental work productivity constraints in the long run, despite positive changes in the family dynamics.

This study has some important merits. First, this study provides valuable insights into the increasing economic inequality of Danish families dealing with child behavior problems even when compared to families with similar socioeconomic outsets. It also indicates that parent-training does not seem to buffer families against this negative trajectory. However, the study offers limited insight into the underlying mechanisms—why and how these patterns emerge—highlighting important questions for future studies into this population, more disadvantaged populations, and in contexts with less robust social security systems than the current. Second, quasi-experimental methods are rare in the field of parent-training and offer new and exciting possibilities for long-term studies in the absence of RCTs and/or follow-up tests, especially in combination with register-based data. While this study design turned out to be less successful in mimicking a causal design, valuable lessons about important criteria for such a design were learned and may help future researchers build stronger designs. A key challenge will be finding useful proxies for child behavior problems—i.e., formal referrals or diagnoses, welfare system referrals, or actual measures of disruptive child behaviors from parents or teachers—to ensure an adequate basis of comparison.

## Conclusion and Implications

In conclusion, families who received the IYPT in a Danish context were economically disadvantaged compared to other Danish families with similar health, demographic, and socioeconomic profiles, and this economic gap widened over time. The most plausible explanation for this widening gap lies not in the intervention itself, but in pre-existing characteristics of the families or their children. These findings highlight the need to recognize that supporting healthy child development—particularly among children with disruptive behaviors—calls for long-term societal strategies. While enhancing parenting practices is important, broader family support that addresses contextual inequalities may also be needed. For instance, increasing the local minimum wage has demonstrated positive effects in socioeconomically disadvantaged settings by reducing economic stress, which is associated with decreases in negative parenting behaviors such as physical and psychological aggression (Schneider et al., 2022), as well as improvement in child behavior outcomes (Woods-Jaeger et al. 2021). Other policy measures, including flexible or supported work schedules and temporary subsidized parental leave, warrant further investigation as potential strategies to buffer families against long-term economic disadvantage.

## Data Availability

The data used for this study were drawn from a restricted access data source and cannot be made publicly available. If a researcher at a university or other research institution outside Denmark wishes to use these data, this may be accomplished by visiting a Danish research institution or by cooperating with researchers working in Denmark. We will provide instructions to any researcher who should wish to replicate our paper.

## Appendix

### Propensity score prediction model

We conducted a binary logistic regression to predict propensity for receiving the IYPT (1 = treatment, 0 = no treatment) based on the following covariates measured at baseline (Time_−3_): number of maternal contacts with the somatic health care system (including in- and outpatient clinic visits, emergency room visits, on call doctor’s contacts, and general physician contacts), maternal age, annual disposable family income in DKK, household type (1 = single parent household, 0 = two-parent household), employment status (1 = maternal unemployment, 0 = maternal employment, retirement, or education), educational level (1 = mother has compulsory education only, 0 = mother has more than compulsory education), and maternal psychiatric history (1 = mother has a formal mental diagnosis related to disruptive behaviors and/or mother has taken prescription medication for attention deficits within the past year, 0 = mother has no formal mental diagnosis related to disruptive behaviors and has not taken prescription medication for attention deficits within the past year).

### Matching results

Seven hundred and seven intervention families were matched with 690 control families. In the matching procedure, propensity scores were calculated for every family across treatment condition, as well as a matching weight accounting for the number of times the family in question has been used as a match. In our analyses, matching weights are applied to appropriately balance the impact of family background characteristics and disposable family income according to their matching characteristics. This is only true for analyses on matched families in our paper.

### Covariate balance diagnostics: see Table 3

**Table 3:** Standardized differences in mean and variance on covariates at baseline (Time_−3_) between intervention and control families after matching

Covariate	Mean	Variance
	St. diff	St. diff
Child age group^a^	.000	
Child gender boy	.039	1.00
Single parent household	.050	1.13
Disposable family income	−.030	0.89
Maternal age	.095	1.41
Maternal somatic health contacts	.038	1.03
Maternal unemployment	.044	1.17
Maternal low educational level	−.027	0.96
Maternal psychiatric history	.003	1.14

Standardized differences in covariate means between treated and untreated individuals are usually considered adequate when ≤|.10| (Markoulidakis et al., 2022). Standardized differences in covariate variance between treated and untreated individuals are usually considered adequate when approaching 1.00 and < 2.00 (Lee & Little, 2017).
